# Identification of Vinculin as a Potential Diagnostic Biomarker for Acute Aortic Dissection Using Label-Free Proteomics

**DOI:** 10.1155/2020/7806409

**Published:** 2020-07-19

**Authors:** He-Qing Wang, Hang Yang, Qian Tang, Yi-Chen Gong, Yuan-Hao Fu, Feng Wan, Bo Yang, Rui Guo, Yong-Liang Zhong, Jun-Ming Zhu, Zhe Zhang

**Affiliations:** ^1^Department of Cardiac Surgery, Peking University Third Hospital, Beijing 100191, China; ^2^Department of Cardiovascular Surgery, Beijing Aortic Disease Center, Beijing Anzhen Hospital, Capital Medical University, Beijing 100020, China

## Abstract

Acute aortic dissection (AAD) is an emergent vascular disease. Currently, its diagnosis depends on clinical and radiological investigations but lacking of serum biomarkers. In this study, we aimed to identify potential serum biomarkers for AAD using label-free proteomics approach. A total of 90 serum samples were collected from three groups: patients with acute aortic dissection (AAD, *n* = 30), patients with acute myocardial infarction (AMI, *n* = 30), and healthy controls (*n* = 30), and the first four samples from each group were selected for label-free proteomics analysis. Using label-free approach, a total of 22 differentially expressed proteins were identified in the serum samples of the AAD group, of which 15 were upregulated and 7 were downregulated as compared to the AMI and healthy control groups. The most prominent increased protein was vinculin, which was selected to validate in total samples. The level of vinculin was significantly elevated in AAD patients (15.8 ng/ml, IQR: 9.3-19.9 ng/ml) than that in AMI patients (8.6 ng/ml, IQR:5.3-11.4 ng/ml) and healthy volunteers (5.3 ng/ml, IQR:2.8-7.6 ng/ml), *P* < 0.0001. Furthermore, the concentration of vinculin both increased in type A and B dissection. At the early stage of AAD, vinculin maintained a high level to 48 hours compared with that of AMI. Our study demonstrated that vinculin may play a role in the early diagnosis of AAD.

## 1. Introduction

Acute aortic dissection (AAD) is the most common thoracic aortic emergency and may be rapidly fatal without early diagnosis and appropriate management [1]. For untreated A type of AAD, the mortality rate increased by 1-2% every 1 hour [2]. Therefore, it is essential to improve the survival rate of AAD by early rapid diagnosis. The main challenge of diagnosis for AAD is to distinguish it from sudden chest pain caused by other diseases (especially acute myocardial infarction, pulmonary embolism, etc.), because the treatment between these patients is completely different or even the opposite, such as when AAD is misdiagnosed as AMI, incorrect use of antiplatelet therapy can increase the risk of further tearing or even rupture of the aorta [3, 4].

Highly sensitive and specific circulating biomarkers are essential for accelerating the diagnosis of AAD. In recent years, markers have been focused on vascular smooth muscle cell injury, including smooth muscle myosin heavy chain, calponin, and creatine kinase isoenzyme BB; extracellular matrix damage, including matrix metalloproteinases, and soluble elastin degradation fragments; and D-dimer released by activation of coagulation and fibrinolysis system [5]. However, none of these biomarkers are widely available with high sensitivity and specificity. Currently, proteomics has become a reliable method for screening early diagnostic markers [6–8]. Compared with traditional absolute isotope labeling (iTRAQ), label-free proteomics can identify more low-abundance proteins which are generally valuable diagnostic biomarkers [9, 10]. With this method, we aim to screen out potential circulating biomarkers which may be released due to the damage of aortic media.

## 2. Materials and Methods

### 2.1. Patients Enrollment

A total of 30 AAD patients, 30 AMI patients, and 30 healthy volunteers were consecutively enrolled from 2018 to 2019 in our hospital. Label-free analysis was performed in the first eight patients (4 AAD and 4 AMI) and four healthy individuals. The time interval of from onset of chest/back/abdominal pain to admission in hospital was less than 48 hours. The diagnosis of AAD was followed by 2014 European guidelines and confirmed by computed tomographic arteriography (CTA) indicating double-lumen sign. AMI was confirmed by elevated electrocardiography (ECG) and cardiac troponin T (cTNT). The protocol was approved by the Ethics Committee of our hospital and all subjects signed informed consent for the study.

10 ml blood samples were collected immediately after admission and centrifuged at 3000 rpm for 10 min at 4°C. The serum was then frozen at −80°C until further tests.

### 2.2. Experimental Methods

#### 2.2.1. Proteomics Technology

14 high abundance proteins in the serum were removed using Human 14 Multiple Affinity Removal System (MARS, agilent, USA). 20 *μ*g of disposed sample was perfused in 4-12% gradient gel (4-12% Bis-TrisNuPAGE gel) and MOPS protein separation buffersystem. The gel was divided into 40 equal fractions and subjected to enzymatic hydrolysis, rinsed with 25 mmol/L sodium hydrogencarbonate, and then dehydrated and dried by acetonitrile. The sample was added with 10 mmol/L dithiothreitol in a water bath at 60°C, alkylated with 50 mmol/L iodoacetamide for 1 h at room temperature, digested by trypsin at 37°C for 4 h, added with 50% acetonitrile/2% formic acid for vacuum centrifuge, and the supernatant was taken for LC-MS/MS analysis. Mass spectrometry was performed by the NanoAcquity HPLC-LTQ-Orbitrap Velos system (AB SCIEX, Canada). The chromatographic column (C18, 50 × 2.1 mm, 1.7 *μ*m) was eluted in a 75 *μ*m analytical column at a flow rate of 350 nl/min. The mobile phase A was an aqueous solution containing 0.1% formic acid, and the mobile phase B was eluted with a gradient of acetonitrile containing 0.1% formic acid. The gradient program was set to 0-3 min 90% B, 3-15 min 90%-40% B, 15-16 min 40%-2% B, 16-18 min 2% B, 18-19 min 2%-90% B, 19-23 min 90% B. The ion source was DuoSpray with electrospray ionization mode for scanning analysis. The negative ion spray voltage was set to -4500 V, and the Turbo spray temperature was 450°C; the positive ion spray voltage was set to 4500 V, and the Turbo spray temperature was 450°C. The precursor ions were scanned by electrostatic orbitrap MS, 15 of which with the highest peaks were selected for MS/MS scan.

#### 2.2.2. Validation of Candidate Biomarkers

D-dimer was tested on a Beckman ACL TOP 700 Automated Coagulation Analyzer by a commercial latex-enhanced immunoturbidimetric assay (HemosIL D-dimer HS, Instrumentation Laboratory, USA). Vinculin was detected using a human ELISA kit (EKU08145, Biomatik, Canada). All procedures were operated in strict accordance with the standard instructions. Duplication of each sample was validated.

### 2.3. Statistical Analysis

The MS raw data were identified in the Swissport Human database by the search software Mascot software (Matrix Science Ltd, USA), and the results were verified by the Scaffold algorithm. 1.5-fold change (FC) of LFQ-intensity between the AAD and control groups was set as the cut off value in differential protein screening, indicating that FC≥1.5 is upregulated and FC≤0.667 is downregulated. The categorical variables were indicated as percentage, and the continuous variables were indicated as mean ± standard deviation or median (interquartile range). Comparative analysis of multiple groups was performed with independent sample *t*-test, one-way analysis of variance, rank sum test, or chi-square test. Statistical significance was defined as *P* < 0.05.

## 3. Results

### 3.1. Clinical Characteristics

30 AAD patients (22 with type A, 8 with type B, 5 with Marfan syndrome, 4 with aortic bicuspid valve malformation), 30 AMI patients, and 30 healthy controls were enrolled (see Table 1). 4 subjects of each group were selected for proteomic analysis. The age of AAD patients was significantly lower than that of the AMI group (48.8 ± 9.9 vs. 63.7 ± 10.8, *P* < 0.0001). Blood sample was collected from patients with AAD and AMI at admission immediately (17.3 ± 10.6 hours vs. 13.5 ± 9.6 hours, *P* = 0.157). There was no significant difference in gender composition among the three groups and in the prevalence of hypertension between AAD and AMI groups.

### 3.2. Proteomics Results and Bioinformatics Analysis

**Figure 1:**
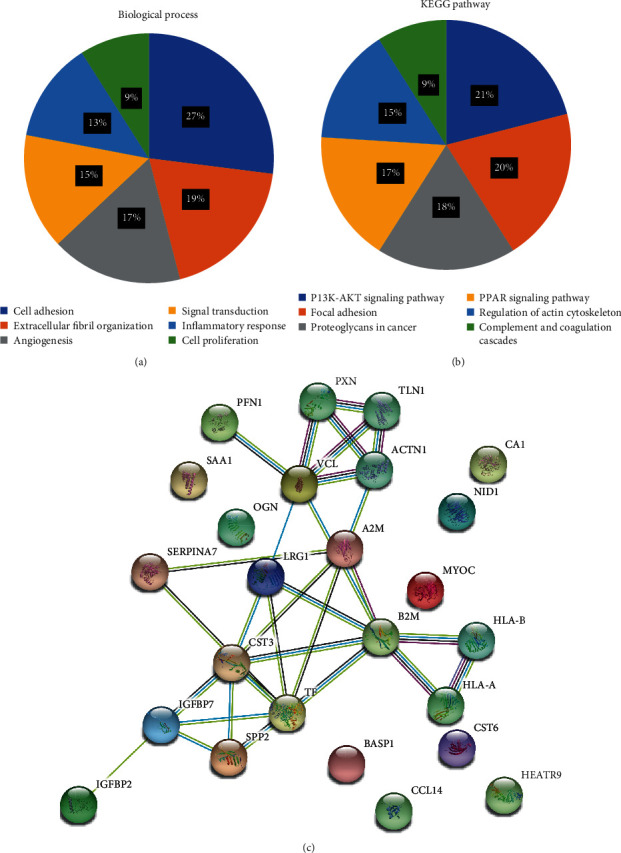
Bioinformatics analysis of the differential proteins. (a) Biological process. (b) Pathway. (c) Network of proteins.

A total of 1096 serum proteins were identified by searching the Swissprot Human database with the Mascot software, and 22 differentially expressed proteins were screened in the serum of the AAD group, 15 of which were upregulated (>1.5 fold) and 7 were downregulated (<0.67 fold) as compared to the AMI and healthy control groups. Based on their biofunction classification, the major biological process types included cell adhesion (27%), extracellular matrix organization (19%), angiogenesis (17%), signal transduction (15%), inflammation response (13%), and cell proliferation (9%) proteins (see Figure 1(a)). A network was constituted by protein-protein interaction analysis on the string website (see Figure 1(c)). In addition, the differentially expressed proteins were involved in P13K-AKT signaling (21%), focal adhesion (20%), proteoglycans in cancer (18%), PPAR signaling (17%), regulation of actin cytoskeleton (15%) and complement, and coagulation cascades (9%) pathways (see Figure 1(b)). Vinculin, a kind of cytoskeletal protein, increased most significantly among the differential proteins (AAD/AMI ratio = 4.85, AAD/Ctl ratio = 8.33) (see Table 2).

### 3.3. Validation of Candidate Biomarkers

**Figure 2:**
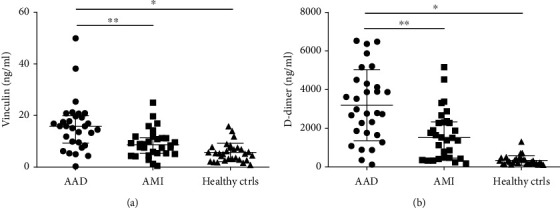
Validation of candidate biomarkers in serum samples. (a) Vinculin. (b) D-dimer. Levels of these candidate biomarkers were measured in serum of AAD (*n* = 30), AMI (*n* = 30), and healthy controls (*n* = 30). Median values are shown by a horizontal line, and the error bars represent the interquartile range of measurements for 30 samples in three groups. *P* values were calculated with Kruskal-Wallis test. ∗*P* < 0.0001, ∗∗*P* < 0.0001.

Based on the proteomics and bioinformatics results, vinculin was selected as a candidate biomarker and validated in total samples; moreover, D-dimer was selected as the gold standard which is recommended in the guideline. The concentration of vinculin (15.8 ng/ml, IQR: 9.3-19.9 ng/ml) was significantly higher in AAD patients than AMI group (8.6 ng/ml, IQR: 5.3-11.4 ng/ml) and healthy control group (5.3 ng/ml, IQR: 2.8-7.6 ng/ml), *P* < 0.0001. Although the concentration of vinculin showed a higher trend in the AMI group compared with the healthy group, the difference was not statistically significant. Meanwhile, the concentration of D-dimer was significantly higher in AAD patients (2886.3 ng/ml, IQR: 1737.2-4362.1) than AMI group (1552.0 ng/ml, IQR465.1-2327.0) and healthy control group (269.5 ng/ml, IQR: 174.3-394.3), *P* < 0.0001 (see Table 3 and Figure 2).

### 3.4. Diagnostic Performance of Candidate Biomarkers

**Figure 3:**
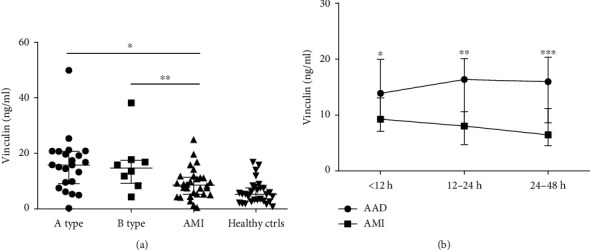
Subanalysis the concentration of vinculin according to type of dissection and time course. (a) Type of dissection (type A = 22, type B = 8). (b) Time windows from onset (0 to 12, 12 to 24, and 24 to 48 hours). Median values are shown by a horizontal line and the error bars represent the interquartile range of measurements in each group. *P* values were calculated with Kruskal-Wallis test. ∗, ∗∗, ∗∗∗*P* < 0.05.

There are reports that some markers may express different concentrations between type A and B dissection due to involvement of different aortic sections [11]; therefore, we further analyzed the concentration of vinculin in different dissection types which demonstrated that the concentration of vinculin was 15.8 ng/ml (IQR: 9.1-20.7) and 14.7 ng/ml (IQR: 9.2-17.5) in type A and B dissection, respectively, both higher than control groups, and no significant difference was discovered between the two dissections (see Figure 3(a)).

Time course was also analyzed from onset to blood collection (time windows of 0 to 12, 12 to 24, and 24 to 48 hours) which showed that the concentration of vinculin increased at an early stage of AAD and maintained a high level to 48 hours compared with that of AMI (see Figure 3(b)).

## 4. Discussion

As a lethal cardiovascular emergency, early rapid diagnosis is essential to improve AAD patient survival [12]. Imaging facilities are pivotal for the diagnosis and surgical treatment of AAD [13]. However, high sensitive and specific AAD biomarkers can help to make the choice of CTA, just similar to troponin of coronary angiography for AMI [14], which can increase the diagnostic pretest probability for the patients with acute chest pain at emergency department. Therefore, biomarkers can shorten the time from admission to surgery. Moreover, for some community hospitals or remote areas which have no facilities, biomarkers can help to decide whether to transfer patients to superior hospital or not.

Proteomics technology has recently become a reliable method for screening disease diagnostic markers [15, 16]. It has been reported that an extracellular matrix protein-lumican was identified by comparing the serum proteomics between the AAD and control groups using iTRAQ technology, but this marker lacked further analysis of its level in different dissection types and time windows [8]. Label-free proteomics, unlike traditional iTRAQ technology, does not depend on isotope labeling and mix of all samples which can accurately reflect individual differences and identifying more low-abundance proteins in serum which contains multiple orders of magnitude abundance proteins [10, 17]. In this study, we first used label-free proteomics to screen for early diagnosis markers of AAD. Based on the previous reports [18], 1.5-fold change (FC) of LFQ-intensity between the AAD and control groups was set as the cut off value in differential protein screening, and 22 differential expressed proteins were identified. Notably, vinculin, a kind of cytoskeletal proteins, increased most significantly among the differential proteins (AAD/AMI ratio = 4.85, AAD/Ctl ratio = 8.33). Further validation of total samples showed that the concentration of vinculin in AAD was 1.8 and 3 times higher than that of the AMI and healthy control groups, respectively.

Different types (A and B) of AAD can present various clinical symptoms, and it is reported that the concentrations of several diagnostic markers were lower in type B dissection than that in the A type (such as TGF-*β*) which limited its availability [19]. In our study, the concentration of vinculin in type A and B dissections showed no significant difference, both higher than that of all controls, indicating that vinculin has a favorable diagnostic performance for both type A and B dissections. In addition, the time course of a biomarker is also a crucial factor, which increases too slowly or decreases too fast will limit its application. Therefore, we analyzed the change of concentration of vinculin from onset which showed it increased rapidly in the early stage (<12 hours) and then maintained at a higher level for 48 hours in AAD patients compared with AMI patients, indicating a satisfactory time window.

It is reported that the aortic wall is fragile in AAD due to media degeneration and elastin degradation, which cannot bear overloaded pressure stimulation, leading to tear of aortic wall [20]. Vinculin is a key protein locating at focal adhesion that regulates mechanochemical pathway of extracellular matrix and cytoskeleton [21, 22], which was found overexpressed in AAD according to our study, may contribute to abnormal migration, adhesion, and proliferation of vascular smooth muscle cells, switching them from contractile phenotype to synthetic phenotype. Synthetic vascular smooth muscle cells can secrete increasing MMPs, leading to imbalance of MMPs and TIMPs, ultimately resulting in proteolysis of elastin in aortic wall [23].

The maintenance of vessel homeostasis relies on the normal contractile function of vascular smooth muscle cells, which is closely related to cell-matrix adhesion. The extracellular mechanical signal (blood stimulation) is converted into intracellular chemical signal via matrix-membrane integrin-focal adhesion pathway, which can further activate the polymerization of downstream actin, stabilize the cytoskeleton, and maintain the plasticity of blood vessel [24]. Therefore, mutations in genes encoding cell-matrix adhesion-related proteins (FBN-1, MFAP5, and COL3A1) can lead to AAD [25]. Our further GO enrichment and KEGG pathway analysis of proteomics results indicated that the most dominant biological process of differential proteins was cell adhesion (27%) in our study, which was consistent with previous proteomic analysis suggesting dysfunction of cell adhesion and matrix remodeling in AAD patients revealing a potential pathophysiological mechanism [18].

D-dimer is the only diagnostic marker in 2014 European guideline (IIa recommendation) [26], which was selected as a comparative marker in our study. The concentration of D-dimer in AAD was nearly 1.8 and 10 times higher than that of the AMI and healthy control groups, respectively, which was consistent with previous reports [27, 28]. D-dimer is the degradation product of thrombus, and its elevation suggests that the activation of blood coagulation and fibrinolysis system generally occurring in DIC, pulmonary embolism, cerebral infarction, myocardial infarction, etc. [28]. In AAD, blood flow enters into the media of aorta through the tear of the intima, activating the coagulation system, thrombosing in false lumen, subsequently fibrin degrading and releasing D-dimer into the blood circulation [29]. Recently, it is reported that D-dimer can play a potential role in activating MMPs [30]. Thus, vinculin and D-dimer may both anticipate the pathophysiology of tissue degradation during AAD via the pathway of MMPs, which is exactly the further investigation purpose in our study.

To our knowledge, the study provides the first evidence that vinculin is associated with AAD and could be a novel biomarker for the early diagnosis. However, there are two limitations in our study. First of all, we did not validate all proteins which were identified in proteomics analysis, especially involved in cell adhesion and focal adhesion. On the other hand, our control groups only enrolled AMI patients but lacking other diseases presenting chest/back pain, especially pulmonary embolism which is easily confused with AAD in differential diagnosis. Therefore, a large prospective cohort should be further validated in suspected patients with AAD to evaluate the clinical application of vinculin.

## 5. Conclusions

A novel biomarker-vinculin was screened in AAD patients compared with AMI patients and healthy controls through label-free proteomics technology. Further validation of all subjects revealed vinculin was elevated significantly. Therefore, the present study will provide meaningful data and new ideas for the differential diagnosis of AAD.

## Figures and Tables

**Table 1:** Clinical characteristics of total subjects.

	AAD	AMI	Healthy Ctrls	*P* value
*n*	30	30	30	
Age (mean ± SD)	48.8 ± 9.9	63.7 ± 10.8	47.3 ± 9.5	0.0001^a^
Gender, male (%)	21 (70.0%)	20 (66.7%)	21 (70.0%)	0.95^b^
Hours from onset to admission (mean ± SD)	17.3 ± 10.6	13.5 ± 9.6	/	0.157^c^
Hypertension (%)	21 (70.0%)	19 (63.3%)	/	0.785^b^
Smoking (%)	9 (30.0%)	10 (33.3)	/	1.000^b^
Type A (%)	22 (73.3%)	/	/	/
Marfan syndrome (%)	5 (15.0)	/	/	/
Bicuspid valve (%)	4 (13.3)	/	/	/
D-dimer (ng/mL) (median, IQR)	2886.3 (1737.2-4362.1)	1552.0 (465.1-2327.0)	269.5 (174.3-394.3)	0.0001^d^

^a^: one-way analysis of variance; ^b^: chi-square test; ^c^: t-test, ^d^: Kruskal-Wallis test, D-dimer was indicated as median (IQR), and the rest of continuous variable were indicated as mean ± SD. AAD: acute aortic dissection; AMI: acute myocardial infarction.

**Table 2:** Differentially expressed proteins (FC>1.5 or <0.667) in AAD identified by label-free proteomics.

Accession	Gene name	Protein name	Biological process	AAD/AMI ratio	AAD/HC ratio
Increased in AAD					
P18206	VCL	Vinculin	Adherens junction assembly	4.85	8.33
P0DJI8	SAA1	Serum amyloid A-1 protein	Activation of MAPK activity	2.36	5.58
Q16627	CCL14	C-C motif chemokine 14	Cellular calcium ion homeostasis	4.61	4.89
P01034	CST3	Cystatin-C	Extracellular fibril organization	2.15	4.47
Q16270	IGFBP7	Insulin-like growth factor-binding protein 7	Cell adhesion	2.12	4.44
Q99972	MYOC	Myocilin	Positive regulation of focal adhesion assembly	3.86	4.14
P18065	IGFBP2	Insulin-like growth factor-binding protein 2	Aging	4.62	3.98
P98160	HSPG2	LG3 peptide	Angiogenesis	3.85	3.76
Q15828	CST6	Cystatin-M	Anatomical structure morphogenesis	2.22	2.96
P20774	OGN	Mimecan	Negative regulation of smooth muscle cell proliferation	3.45	2.79
P07737	PFN1	Profilin-1	Actin cytoskeleton organization	2.95	2.48
P80723	BASP1	Brain acid soluble protein 1	Diaphragm development	1.57	2.45
P02750	LRG1	Leucine-rich alpha-2-glycoprotein	Brown fat cell differentiation	1.74	2.36
P61769	B2M	Beta-2-microglobulin	Antibacterial humoral response	2.24	2.06
P14543	NID1	Nidogen-1	Basement membrane organization	1.53	1.73
Decreased in AAD					
Q13103	SPP2	Secreted phosphoprotein 24	Bone remodeling	0.51	0.24
P00915	CA1	Carbonic anhydrase 1	Bicarbonate transport	0.27	0.29
P05543	SERPINA7	Thyroxine-binding globulin	Thyroid hormone transport	0.24	0.33
P01871	IGHM	Ig mu chain C region	Adaptive immune response	0.58	0.42
A2RTY3	HEATR9	Protein HEATR9	Hematopoietic progenitor cell differentiation	0.54	0.45
P02787	TF	Serotransferrin	Cellular iron ion homeostasis	0.42	0.47
P01023	A2M	Alpha-2-macroglobulin	Blood coagulation, intrinsic pathway	0.46	0.56

AAD: acute aortic dissection; AMI: acute myocardial infarction.

**Table 3:** The serum concentrations of vinculin and D-dimer in each group.

	AAD (*n* = 30)	AMI (*n* = 30)	HC (*n* = 30)	*P* value
Vinculin (ng/mL)^a^	15.8 (9.3-19.9)	8.6 (5.3-11.4)	5.29 (2.8-7.6)	<0.0001^b^
D-dimer(ng/mL)^a^	2886.3 (1737.2-4362.1)	1552.0 (465.1-2327.0)	269.5 (174.3-394.3)	<0.0001^b^

^a^: median (interquartile range); ^b^: Kruskal-Wallis test. AAD: acute aortic dissection; AMI: acute myocardial infarction; HC: healthy controls.

## Data Availability

The data used to support the findings of this study are available from the corresponding author upon request.
